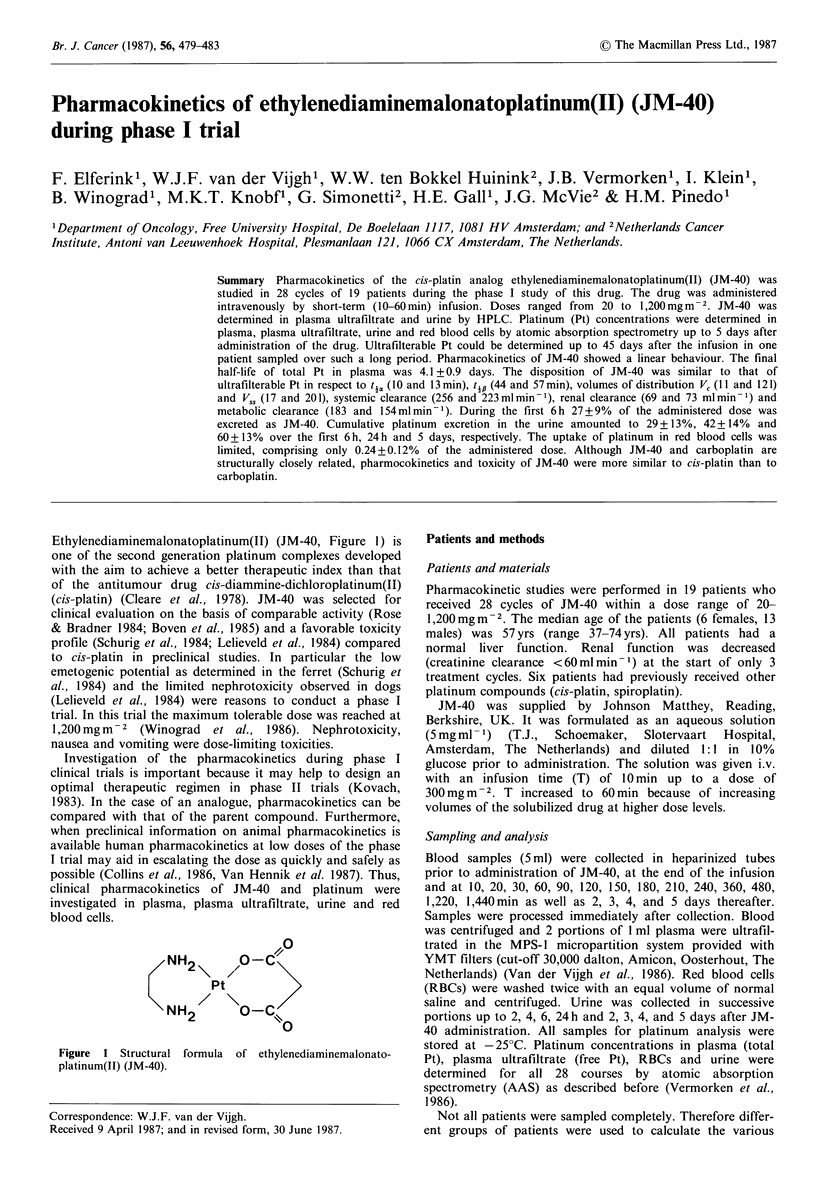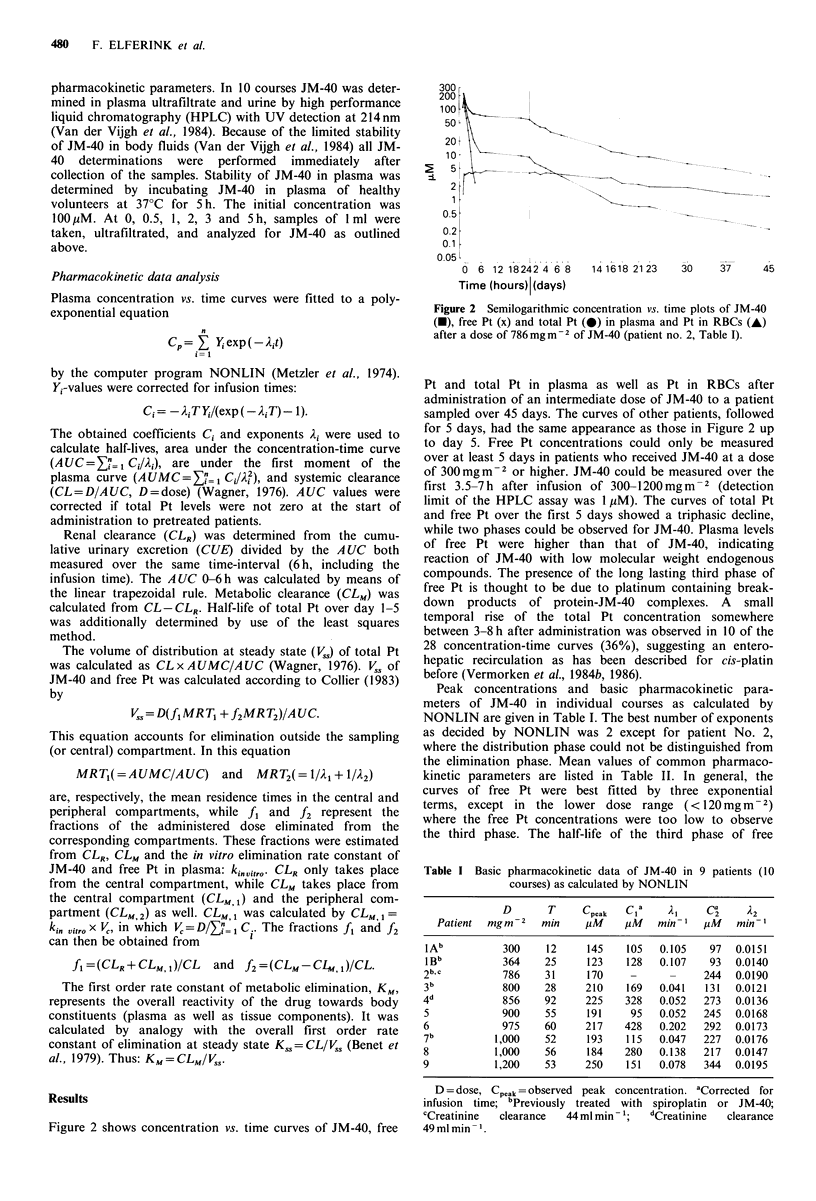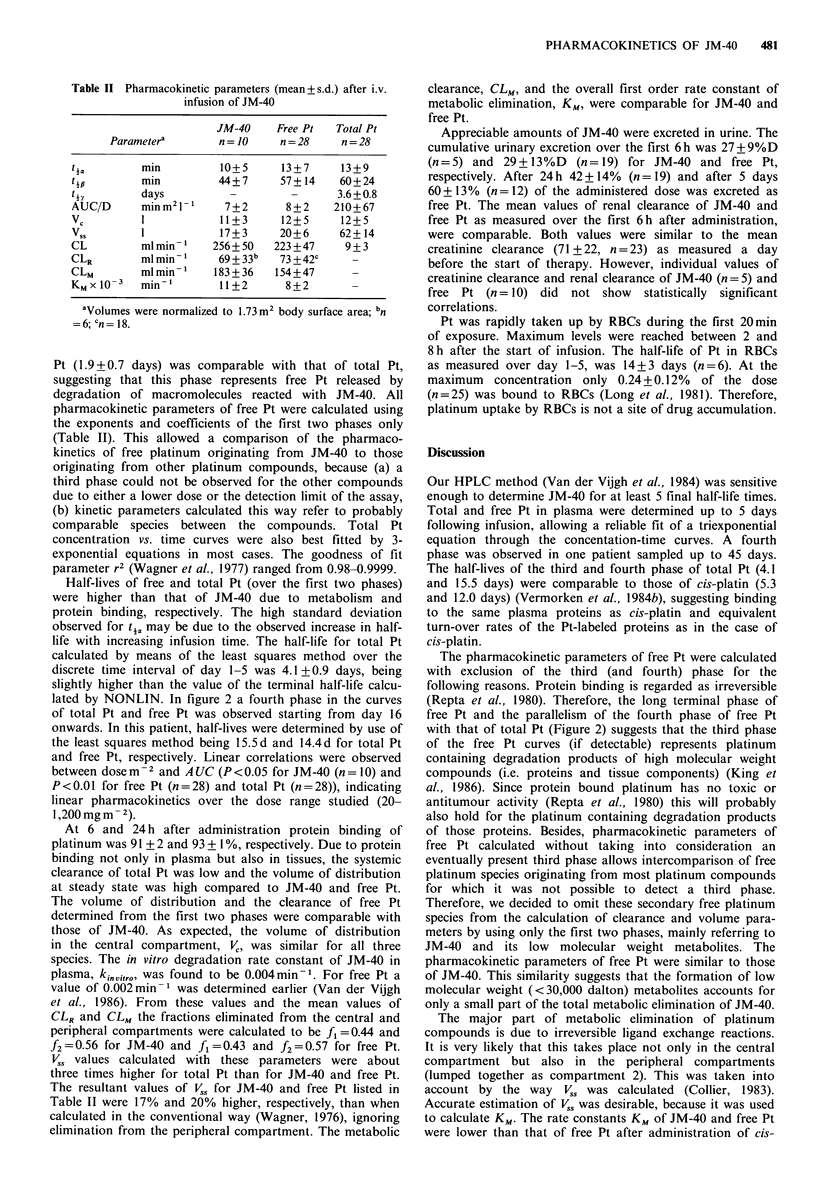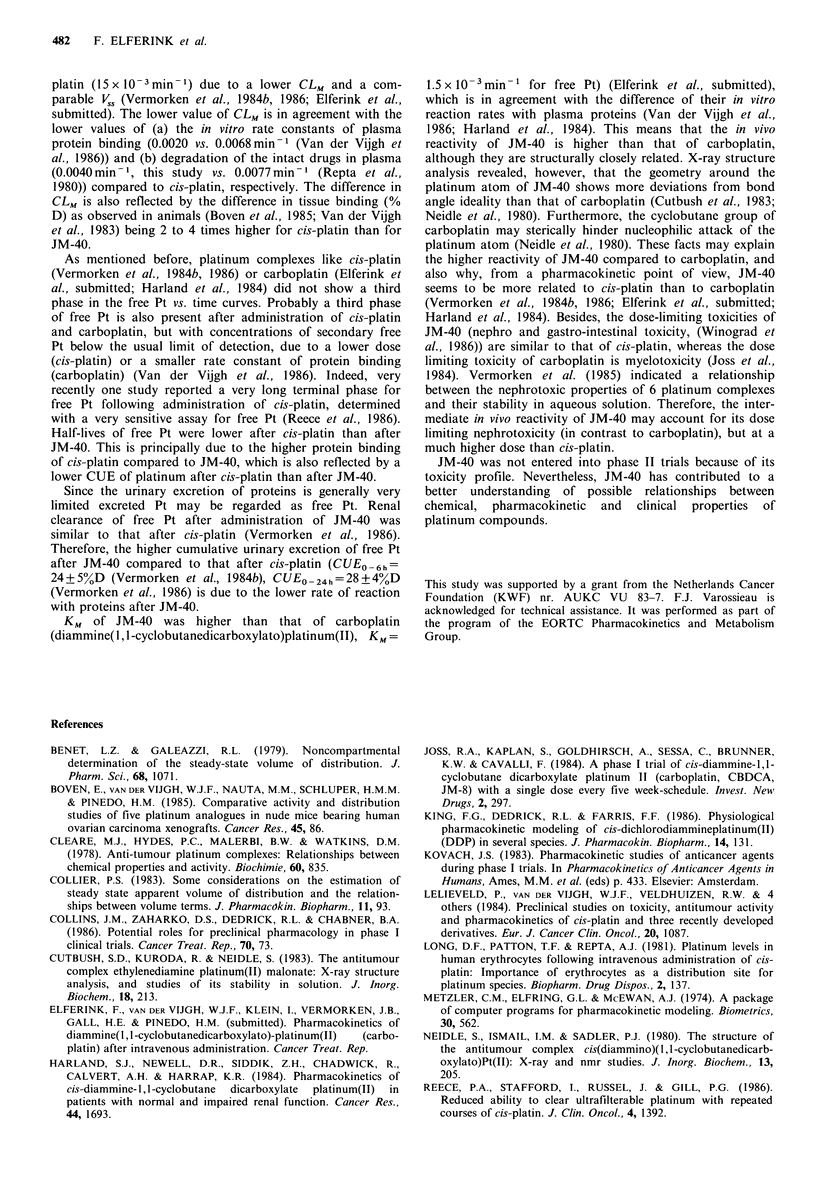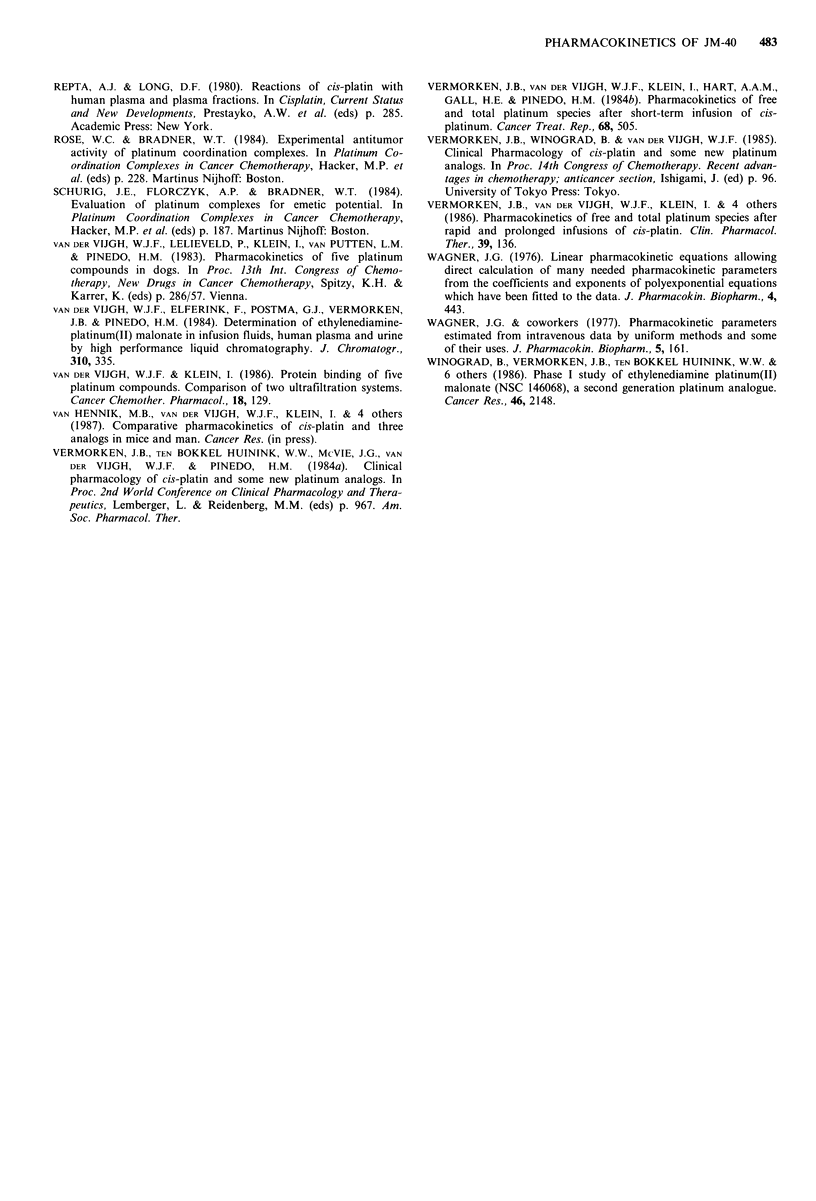# Pharmacokinetics of ethylenediaminemalonatoplatinum(II) (JM-40) during phase I trial.

**DOI:** 10.1038/bjc.1987.228

**Published:** 1987-10

**Authors:** F. Elferink, W. J. van der Vijgh, W. W. ten Bokkel Huinink, J. B. Vermorken, I. Klein, B. Winograd, M. K. Knobf, G. Simonetti, H. E. Gall, J. G. McVie

**Affiliations:** Department of Oncology, Free University Hospital, Amsterdam, The Netherlands.

## Abstract

Pharmacokinetics of the cis-platin analog ethylenediaminemalonatoplatinum(II) (JM-410) was studied in 28 cycles of 19 patients during the phase I study of this drug. The drug was administered intravenously by short-term (10-60 min) infusion. Doses ranged from 20 to 1,200mg m-2. JM-40 was determined in plasma ultrafiltrate and urine by HPLC. Platinum (Pt) concentrations were determined in plasma, plasma ultrafiltrate, urine and red blood cells by atomic absorption spectrometry up to 5 days after administration of the drug. Ultrafilterable Pt could be determined up to 45 days after the infusion in one patient sampled over such a long period. Pharmacokinetics of JM-40 showed a linear behaviour. The final half-life of total Pt in plasma was 4.1 +/- 0.9 days. The disposition of JM-40 was similar to that of ultrafilterable Pt in respect to t1/2 alpha (10 and 13 min), t1/2 beta (44 and 57 min), volumes of distribution Vc (11 and 121) and Vss (17 and 201), systemic clearance (256 and 223 ml min-1), renal clearance (69 and 73 ml min-1) and metabolic clearance (183 and 154 ml min-1). During the first 6 h 27 +/- 9% of the administered dose was excreted as JM-40. Cumulative platinum excretion in the urine amounted to 29 +/- 13% and 60 +/- 13% over the first 6 h, 24 h and 5 days, respectively. The uptake of platinum in red blood cells was limited, comprising only 0.24 +/- 0.12% of the administered dose. Although JM-40 and carboplatin are structurally closely related, pharmocokinetics and toxicity of JM-40 were more similar to cis-platin than to carboplatin.


*Br. J. Cancer* (1987), **56**, 479–483 © The Macmillan Press Ltd., 1987

### Pharmacokinetics of ethylenediaminemalonatoplatinum(II) (JM-40) during phase I trial

F. Elferink[1], W.J.F. van der Vijgh[1], W.W. ten Bokkel Huinink[2], J.B. Vermorken[1], I. Klein[1], B. Winograd[1], M.K.T. Knobf[1], G. Simonetti[2], H.E. Gall[1], J.G. McVie[2] & H.M. Pinedo[1]

[1]*Department of Oncology, Free University Hospital, De Boelelaan 1117, 1081 HV Amsterdam; and* [2]*Netherlands Cancer Institute, Antoni van Leeuwenhoek Hospital, Plesmanlaan 121, 1066 CX Amsterdam, The Netherlands.*

**Summary** Pharmacokinetics of the *cis*-platin analog ethylenediaminemalonatoplatinum(II) (JM-40) was studied in 28 cycles of 19 patients during the phase I study of this drug. The drug was administered intravenously by short-term (10–60 min) infusion. Doses ranged from 20 to 1,200 mg m$^{-2}$. JM-40 was determined in plasma ultrafiltrate and urine by HPLC. Platinum (Pt) concentrations were determined in plasma, plasma ultrafiltrate, urine and red blood cells by atomic absorption spectrometry up to 5 days after administration of the drug. Ultrafilterable Pt could be determined up to 45 days after the infusion in one patient sampled over such a long period. Pharmacokinetics of JM-40 showed a linear behaviour. The final half-life of total Pt in plasma was 4.1±0.9 days. The disposition of JM-40 was similar to that of ultrafilterable Pt in respect to $t_{\frac{1}{2}\alpha}$ (10 and 13 min), $t_{\frac{1}{2}\beta}$ (44 and 57 min), volumes of distribution $V_c$ (11 and 12 l) and $V_{ss}$ (17 and 20 l), systemic clearance (256 and 223 ml min$^{-1}$), renal clearance (69 and 73 ml min$^{-1}$) and metabolic clearance (183 and 154 ml min$^{-1}$). During the first 6 h 27±9% of the administered dose was excreted as JM-40. Cumulative platinum excretion in the urine amounted to 29±13%, 42±14% and 60±13% over the first 6 h, 24 h and 5 days, respectively. The uptake of platinum in red blood cells was limited, comprising only 0.24±0.12% of the administered dose. Although JM-40 and carboplatin are structurally closely related, pharmocokinetics and toxicity of JM-40 were more similar to *cis*-platin than to carboplatin.

Ethylenediaminemalonatoplatinum(II) (JM-40, Figure 1) is one of the second generation platinum complexes developed with the aim to achieve a better therapeutic index than that of the antitumour drug *cis*-diammine-dichloroplatinum(II) (*cis*-platin) (Cleare *et al.*, 1978). JM-40 was selected for clinical evaluation on the basis of comparable activity (Rose & Bradner 1984; Boven *et al.*, 1985) and a favorable toxicity profile (Schurig *et al.*, 1984; Lelieveld *et al.*, 1984) compared to *cis*-platin in preclinical studies. In particular the low emetogenic potential as determined in the ferret (Schurig *et al.*, 1984) and the limited nephrotoxicity observed in dogs (Lelieveld *et al.*, 1984) were reasons to conduct a phase I trial. In this trial the maximum tolerable dose was reached at 1,200 mg m$^{-2}$ (Winograd *et al.*, 1986). Nephrotoxicity, nausea and vomiting were dose-limiting toxicities.

Investigation of the pharmacokinetics during phase I clinical trials is important because it may help to design an optimal therapeutic regimen in phase II trials (Kovach, 1983). In the case of an analogue, pharmacokinetics can be compared with that of the parent compound. Furthermore, when preclinical information on animal pharmacokinetics is available human pharmacokinetics at low doses of the phase I trial may aid in escalating the dose as quickly and safely as possible (Collins *et al.*, 1986, Van Hennik *et al.* 1987). Thus, clinical pharmacokinetics of JM-40 and platinum were investigated in plasma, plasma ultrafiltrate, urine and red blood cells.

**Figure 1** Structural formula of ethylenediaminemalonatoplatinum(II) (JM-40).

Correspondence: W.J.F. van der Vijgh.
Received 9 April 1987; and in revised form, 30 June 1987.

### Patients and methods

### *Patients and materials*

Pharmacokinetic studies were performed in 19 patients who received 28 cycles of JM-40 within a dose range of 20–1,200 mg m$^{-2}$. The median age of the patients (6 females, 13 males) was 57 yrs (range 37–74 yrs). All patients had a normal liver function. Renal function was decreased (creatinine clearance <60 ml min$^{-1}$) at the start of only 3 treatment cycles. Six patients had previously received other platinum compounds (*cis*-platin, spiroplatin).

JM-40 was supplied by Johnson Matthey, Reading, Berkshire, UK. It was formulated as an aqueous solution (5 mg ml$^{-1}$) (T.J., Schoemaker, Slotervaart Hospital, Amsterdam, The Netherlands) and diluted 1:1 in 10% glucose prior to administration. The solution was given i.v. with an infusion time (T) of 10 min up to a dose of 300 mg m$^{-2}$. T increased to 60 min because of increasing volumes of the solubilized drug at higher dose levels.

### *Sampling and analysis*

Blood samples (5 ml) were collected in heparinized tubes prior to administration of JM-40, at the end of the infusion and at 10, 20, 30, 60, 90, 120, 150, 180, 210, 240, 360, 480, 1,220, 1,440 min as well as 2, 3, 4, and 5 days thereafter. Samples were processed immediately after collection. Blood was centrifuged and 2 portions of 1 ml plasma were ultrafiltrated in the MPS-1 micropartition system provided with YMT filters (cut-off 30,000 dalton, Amicon, Oosterhout, The Netherlands) (Van der Vijgh *et al.*, 1986). Red blood cells (RBCs) were washed twice with an equal volume of normal saline and centrifuged. Urine was collected in successive portions up to 2, 4, 6, 24 h and 2, 3, 4, and 5 days after JM-40 administration. All samples for platinum analysis were stored at −25°C. Platinum concentrations in plasma (total Pt), plasma ultrafiltrate (free Pt), RBCs and urine were determined for all 28 courses by atomic absorption spectrometry (AAS) as described before (Vermorken *et al.*, 1986).

Not all patients were sampled completely. Therefore different groups of patients were used to calculate the various pharmacokinetic parameters. In 10 courses JM-40 was determined in plasma ultrafiltrate and urine by high performance liquid chromatography (HPLC) with UV detection at 214 nm (Van der Vijgh *et al.*, 1984). Because of the limited stability of JM-40 in body fluids (Van der Vijgh *et al.*, 1984) all JM-40 determinations were performed immediately after collection of the samples. Stability of JM-40 in plasma was determined by incubating JM-40 in plasma of healthy volunteers at 37°C for 5 h. The initial concentration was 100 $\mu$M. At 0, 0.5, 1, 2, 3 and 5 h, samples of 1 ml were taken, ultrafiltrated, and analyzed for JM-40 as outlined above.

*Pharmacokinetic data analysis*

Plasma concentration *vs.* time curves were fitted to a polyexponential equation

$$C_p = \sum_{i=1}^{n} Y_i \exp(-\lambda_i t)$$

by the computer program NONLIN (Metzler *et al.*, 1974). $Y_i$-values were corrected for infusion times:

$$C_i = -\lambda_i T Y_i / (\exp(-\lambda_i T) - 1).$$

The obtained coefficients $C_i$ and exponents $\lambda_i$ were used to calculate half-lives, area under the concentration-time curve ($AUC = \sum_{i=1}^{n} C_i/\lambda_i$), are under the first moment of the plasma curve ($AUMC = \sum_{i=1}^{n} C_i/\lambda_i^2$), and systemic clearance ($CL = D/AUC$, $D$ = dose) (Wagner, 1976). $AUC$ values were corrected if total Pt levels were not zero at the start of administration to pretreated patients.

Renal clearance ($CL_R$) was determined from the cumulative urinary excretion ($CUE$) divided by the $AUC$ both measured over the same time-interval (6 h, including the infusion time). The $AUC$ 0–6 h was calculated by means of the linear trapezoidal rule. Metabolic clearance ($CL_M$) was calculated from $CL - CL_R$. Half-life of total Pt over day 1–5 was additionally determined by use of the least squares method.

The volume of distribution at steady state ($V_{ss}$) of total Pt was calculated as $CL \times AUMC/AUC$ (Wagner, 1976). $V_{ss}$ of JM-40 and free Pt was calculated according to Collier (1983) by

$$V_{ss} = D(f_1 MRT_1 + f_2 MRT_2)/AUC.$$

This equation accounts for elimination outside the sampling (or central) compartment. In this equation

$$MRT_1 (= AUMC/AUC) \quad \text{and} \quad MRT_2 (= 1/\lambda_1 + 1/\lambda_2)$$

are, respectively, the mean residence times in the central and peripheral compartments, while $f_1$ and $f_2$ represent the fractions of the administered dose eliminated from the corresponding compartments. These fractions were estimated from $CL_R$, $CL_M$ and the *in vitro* elimination rate constant of JM-40 and free Pt in plasma: $k_{in\,vitro}$. $CL_R$ only takes place from the central compartment, while $CL_M$ takes place from the central compartment ($CL_{M,1}$) and the peripheral compartment ($CL_{M,2}$) as well. $CL_{M,1}$ was calculated by $CL_{M,1} = k_{in\,vitro} \times V_c$, in which $V_c = D/\sum_{i=1}^{n} C_i$. The fractions $f_1$ and $f_2$ can then be obtained from

$$f_1 = (CL_R + CL_{M,1})/CL \quad \text{and} \quad f_2 = (CL_M - CL_{M,1})/CL.$$

The first order rate constant of metabolic elimination, $K_M$, represents the overall reactivity of the drug towards body constituents (plasma as well as tissue components). It was calculated by analogy with the overall first order rate constant of elimination at steady state $K_{ss} = CL/V_{ss}$ (Benet *et al.*, 1979). Thus: $K_M = CL_M/V_{ss}$.

### Results

Figure 2 shows concentration *vs.* time curves of JM-40, free Pt and total Pt in plasma as well as Pt in RBCs after administration of an intermediate dose of JM-40 to a patient sampled over 45 days. The curves of other patients, followed for 5 days, had the same appearance as those in Figure 2 up to day 5. Free Pt concentrations could only be measured over at least 5 days in patients who received JM-40 at a dose of 300 mg m$^{-2}$ or higher. JM-40 could be measured over the first 3.5–7 h after infusion of 300–1200 mg m$^{-2}$ (detection limit of the HPLC assay was 1 $\mu$M). The curves of total Pt and free Pt over the first 5 days showed a triphasic decline, while two phases could be observed for JM-40. Plasma levels of free Pt were higher than that of JM-40, indicating reaction of JM-40 with low molecular weight endogenous compounds. The presence of the long lasting third phase of free Pt is thought to be due to platinum containing breakdown products of protein-JM-40 complexes. A small temporal rise of the total Pt concentration somewhere between 3–8 h after administration was observed in 10 of the 28 concentration-time curves (36%), suggesting an enterohepatic recirculation as has been described for *cis*-platin before (Vermorken *et al.*, 1984*b*, 1986).

**Figure 2** Semilogarithmic concentration *vs.* time plots of JM-40 (■), free Pt (x) and total Pt (●) in plasma and Pt in RBCs (▲) after a dose of 786 mg m$^{-2}$ of JM-40 (patient no. 2, Table I).

Peak concentrations and basic pharmacokinetic parameters of JM-40 in individual courses as calculated by NONLIN are given in Table I. The best number of exponents as decided by NONLIN was 2 except for patient No. 2, where the distribution phase could not be distinguished from the elimination phase. Mean values of common pharmacokinetic parameters are listed in Table II. In general, the curves of free Pt were best fitted by three exponential terms, except in the lower dose range (< 120 mg m$^{-2}$) where the free Pt concentrations were too low to observe the third phase. The half-life of the third phase of free

**Table I** Basic pharmacokinetic data of JM-40 in 9 patients (10 courses) as calculated by NONLIN

| *Patient* | *D* mg m$^{-2}$ | *T* min | $C_{peak}$ $\mu M$ | $C_1$[a] $\mu M$ | $\lambda_1$ min$^{-1}$ | $C_2^a$ $\mu M$ | $\lambda_2$ min$^{-1}$ |
|---|---|---|---|---|---|---|---|
| 1A[b] | 300 | 12 | 145 | 105 | 0.105 | 97 | 0.0151 |
| 1B[b] | 364 | 25 | 123 | 128 | 0.107 | 93 | 0.0140 |
| 2[b,c] | 786 | 31 | 170 | – | – | 244 | 0.0190 |
| 3[b] | 800 | 28 | 210 | 169 | 0.041 | 131 | 0.0121 |
| 4[d] | 856 | 92 | 225 | 328 | 0.052 | 273 | 0.0136 |
| 5 | 900 | 55 | 191 | 95 | 0.052 | 245 | 0.0168 |
| 6 | 975 | 60 | 217 | 428 | 0.202 | 292 | 0.0173 |
| 7[b] | 1,000 | 52 | 193 | 115 | 0.047 | 227 | 0.0176 |
| 8 | 1,000 | 56 | 184 | 280 | 0.138 | 217 | 0.0147 |
| 9 | 1,200 | 53 | 250 | 151 | 0.078 | 344 | 0.0195 |

D = dose, $C_{peak}$ = observed peak concentration. [a]Corrected for infusion time; [b]Previously treated with spiroplatin or JM-40; [c]Creatinine clearance 44 ml min$^{-1}$; [d]Creatinine clearance 49 ml min$^{-1}$.

**Table II** Pharmacokinetic parameters (mean ± s.d.) after i.v. infusion of JM-40

| Parameter[a] | | *JM-40* *n*=*10* | *Free Pt* *n*=*28* | *Total Pt* *n*=*28* |
|---|---|---|---|---|
| $t_{\frac{1}{2}\alpha}$ | min | 10±5 | 13±7 | 13±9 |
| $t_{\frac{1}{2}\beta}$ | min | 44±7 | 57±14 | 60±24 |
| $t_{\frac{1}{2}\gamma}$ | days | – | – | 3.6±0.8 |
| AUC/D | $\text{min m}^2\text{l}^{-1}$ | 7±2 | 8±2 | 210±67 |
| $V_c$ | l | 11±3 | 12±5 | 12±5 |
| $V_{ss}$ | l | 17±3 | 20±6 | 62±14 |
| CL | $\text{ml min}^{-1}$ | 256±50 | 223±47 | 9±3 |
| $CL_R$ | $\text{ml min}^{-1}$ | 69±33[b] | 73±42[c] | – |
| $CL_M$ | $\text{ml min}^{-1}$ | 183±36 | 154±47 | – |
| $K_M \times 10^{-3}$ | $\text{min}^{-1}$ | 11±2 | 8±2 | – |

[a]Volumes were normalized to $1.73\,\text{m}^2$ body surface area; [b]$n=6$; [c]$n=18$.

Pt (1.9±0.7 days) was comparable with that of total Pt, suggesting that this phase represents free Pt released by degradation of macromolecules reacted with JM-40. All pharmacokinetic parameters of free Pt were calculated using the exponents and coefficients of the first two phases only (Table II). This allowed a comparison of the pharmacokinetics of free platinum originating from JM-40 to those originating from other platinum compounds, because (a) a third phase could not be observed for the other compounds due to either a lower dose or the detection limit of the assay, (b) kinetic parameters calculated this way refer to probably comparable species between the compounds. Total Pt concentration *vs.* time curves were also best fitted by 3-exponential equations in most cases. The goodness of fit parameter $r^2$ (Wagner *et al.*, 1977) ranged from 0.98–0.9999.

Half-lives of free and total Pt (over the first two phases) were higher than that of JM-40 due to metabolism and protein binding, respectively. The high standard deviation observed for $t_{\frac{1}{2}\alpha}$ may be due to the observed increase in half-life with increasing infusion time. The half-life for total Pt calculated by means of the least squares method over the discrete time interval of day 1–5 was 4.1±0.9 days, being slightly higher than the value of the terminal half-life calculated by NONLIN. In figure 2 a fourth phase in the curves of total Pt and free Pt was observed starting from day 16 onwards. In this patient, half-lives were determined by use of the least squares method being 15.5 d and 14.4 d for total Pt and free Pt, respectively. Linear correlations were observed between dose $\text{m}^{-2}$ and *AUC* ($P<0.05$ for JM-40 ($n=10$) and $P<0.01$ for free Pt ($n=28$) and total Pt ($n=28$)), indicating linear pharmacokinetics over the dose range studied (20–1,200 $\text{mg m}^{-2}$).

At 6 and 24 h after administration protein binding of platinum was 91±2 and 93±1%, respectively. Due to protein binding not only in plasma but also in tissues, the systemic clearance of total Pt was low and the volume of distribution at steady state was high compared to JM-40 and free Pt. The volume of distribution and the clearance of free Pt determined from the first two phases were comparable with those of JM-40. As expected, the volume of distribution in the central compartment, $V_c$, was similar for all three species. The *in vitro* degradation rate constant of JM-40 in plasma, $k_{in\,vitro}$, was found to be 0.004 $\text{min}^{-1}$. For free Pt a value of 0.002 $\text{min}^{-1}$ was determined earlier (Van der Vijgh *et al.*, 1986). From these values and the mean values of $CL_R$ and $CL_M$ the fractions eliminated from the central and peripheral compartments were calculated to be $f_1=0.44$ and $f_2=0.56$ for JM-40 and $f_1=0.43$ and $f_2=0.57$ for free Pt. $V_{ss}$ values calculated with these parameters were about three times higher for total Pt than for JM-40 and free Pt. The resultant values of $V_{ss}$ for JM-40 and free Pt listed in Table II were 17% and 20% higher, respectively, than when calculated in the conventional way (Wagner, 1976), ignoring elimination from the peripheral compartment. The metabolic clearance, $CL_M$, and the overall first order rate constant of metabolic elimination, $K_M$, were comparable for JM-40 and free Pt.

Appreciable amounts of JM-40 were excreted in urine. The cumulative urinary excretion over the first 6 h was 27±9%D ($n=5$) and 29±13%D ($n=19$) for JM-40 and free Pt, respectively. After 24 h 42±14% ($n=19$) and after 5 days 60±13% ($n=12$) of the administered dose was excreted as free Pt. The mean values of renal clearance of JM-40 and free Pt as measured over the first 6 h after administration, were comparable. Both values were similar to the mean creatinine clearance (71±22, $n=23$) as measured a day before the start of therapy. However, individual values of creatinine clearance and renal clearance of JM-40 ($n=5$) and free Pt ($n=10$) did not show statistically significant correlations.

Pt was rapidly taken up by RBCs during the first 20 min of exposure. Maximum levels were reached between 2 and 8 h after the start of infusion. The half-life of Pt in RBCs as measured over day 1–5, was 14±3 days ($n=6$). At the maximum concentration only 0.24±0.12% of the dose ($n=25$) was bound to RBCs (Long *et al.*, 1981). Therefore, platinum uptake by RBCs is not a site of drug accumulation.

### Discussion

Our HPLC method (Van der Vijgh *et al.*, 1984) was sensitive enough to determine JM-40 for at least 5 final half-life times. Total and free Pt in plasma were determined up to 5 days following infusion, allowing a reliable fit of a triexponential equation through the concentation-time curves. A fourth phase was observed in one patient sampled up to 45 days. The half-lives of the third and fourth phase of total Pt (4.1 and 15.5 days) were comparable to those of *cis*-platin (5.3 and 12.0 days) (Vermorken *et al.*, 1984*b*), suggesting binding to the same plasma proteins as *cis*-platin and equivalent turn-over rates of the Pt-labeled proteins as in the case of *cis*-platin.

The pharmacokinetic parameters of free Pt were calculated with exclusion of the third (and fourth) phase for the following reasons. Protein binding is regarded as irreversible (Repta *et al.*, 1980). Therefore, the long terminal phase of free Pt and the parallelism of the fourth phase of free Pt with that of total Pt (Figure 2) suggests that the third phase of the free Pt curves (if detectable) represents platinum containing degradation products of high molecular weight compounds (i.e. proteins and tissue components) (King *et al.*, 1986). Since protein bound platinum has no toxic or antitumour activity (Repta *et al.*, 1980) this will probably also hold for the platinum containing degradation products of those proteins. Besides, pharmacokinetic parameters of free Pt calculated without taking into consideration an eventually present third phase allows intercomparison of free platinum species originating from most platinum compounds for which it was not possible to detect a third phase. Therefore, we decided to omit these secondary free platinum species from the calculation of clearance and volume parameters by using only the first two phases, mainly referring to JM-40 and its low molecular weight metabolites. The pharmacokinetic parameters of free Pt were similar to those of JM-40. This similarity suggests that the formation of low molecular weight (<30,000 dalton) metabolites accounts for only a small part of the total metabolic elimination of JM-40.

The major part of metabolic elimination of platinum compounds is due to irreversible ligand exchange reactions. It is very likely that this takes place not only in the central compartment but also in the peripheral compartments (lumped together as compartment 2). This was taken into account by the way $V_{ss}$ was calculated (Collier, 1983). Accurate estimation of $V_{ss}$ was desirable, because it was used to calculate $K_M$. The rate constants $K_M$ of JM-40 and free Pt were lower than that of free Pt after administration of *cis*-

platin ($15 \times 10^{-3} min^{-1}$) due to a lower $CL_M$ and a comparable $V_{ss}$ (Vermorken *et al.*, 1984*b*, 1986; Elferink *et al.*, submitted). The lower value of $CL_M$ is in agreement with the lower values of (a) the *in vitro* rate constants of plasma protein binding (0.0020 *vs.* 0.0068 $min^{-1}$ (Van der Vijgh *et al.*, 1986)) and (b) degradation of the intact drugs in plasma (0.0040 $min^{-1}$, this study *vs.* 0.0077 $min^{-1}$ (Repta *et al.*, 1980)) compared to *cis*-platin, respectively. The difference in $CL_M$ is also reflected by the difference in tissue binding (% D) as observed in animals (Boven *et al.*, 1985; Van der Vijgh *et al.*, 1983) being 2 to 4 times higher for *cis*-platin than for JM-40.

As mentioned before, platinum complexes like *cis*-platin (Vermorken *et al.*, 1984*b*, 1986) or carboplatin (Elferink *et al.*, submitted; Harland *et al.*, 1984) did not show a third phase in the free Pt *vs.* time curves. Probably a third phase of free Pt is also present after administration of *cis*-platin and carboplatin, but with concentrations of secondary free Pt below the usual limit of detection, due to a lower dose (*cis*-platin) or a smaller rate constant of protein binding (carboplatin) (Van der Vijgh *et al.*, 1986). Indeed, very recently one study reported a very long terminal phase for free Pt following administration of *cis*-platin, determined with a very sensitive assay for free Pt (Reece *et al.*, 1986). Half-lives of free Pt were lower after *cis*-platin than after JM-40. This is principally due to the higher protein binding of *cis*-platin compared to JM-40, which is also reflected by a lower CUE of platinum after *cis*-platin than after JM-40.

Since the urinary excretion of proteins is generally very limited excreted Pt may be regarded as free Pt. Renal clearance of free Pt after administration of JM-40 was similar to that after *cis*-platin (Vermorken *et al.*, 1986). Therefore, the higher cumulative urinary excretion of free Pt after JM-40 compared to that after *cis*-platin ($CUE_{0-6h}$ = 24 ± 5%D (Vermorken *et al.*, 1984*b*), $CUE_{0-24h}$ = 28 ± 4%D (Vermorken *et al.*, 1986) is due to the lower rate of reaction with proteins after JM-40.

$K_M$ of JM-40 was higher than that of carboplatin (diammine(1,1-cyclobutanedicarboxylato)platinum(II), $K_M$ = $1.5 \times 10^{-3} min^{-1}$ for free Pt) (Elferink *et al.*, submitted), which is in agreement with the difference of their *in vitro* reaction rates with plasma proteins (Van der Vijgh *et al.*, 1986; Harland *et al.*, 1984). This means that the *in vivo* reactivity of JM-40 is higher than that of carboplatin, although they are structurally closely related. X-ray structure analysis revealed, however, that the geometry around the platinum atom of JM-40 shows more deviations from bond angle ideality than that of carboplatin (Cutbush *et al.*, 1983; Neidle *et al.*, 1980). Furthermore, the cyclobutane group of carboplatin may sterically hinder nucleophilic attack of the platinum atom (Neidle *et al.*, 1980). These facts may explain the higher reactivity of JM-40 compared to carboplatin, and also why, from a pharmacokinetic point of view, JM-40 seems to be more related to *cis*-platin than to carboplatin (Vermorken *et al.*, 1984*b*, 1986; Elferink *et al.*, submitted; Harland *et al.*, 1984). Besides, the dose-limiting toxicities of JM-40 (nephro and gastro-intestinal toxicity, (Winograd *et al.*, 1986)) are similar to that of *cis*-platin, whereas the dose limiting toxicity of carboplatin is myelotoxicity (Joss *et al.*, 1984). Vermorken *et al.* (1985) indicated a relationship between the nephrotoxic properties of 6 platinum complexes and their stability in aqueous solution. Therefore, the intermediate *in vivo* reactivity of JM-40 may account for its dose limiting nephrotoxicity (in contrast to carboplatin), but at a much higher dose than *cis*-platin.

JM-40 was not entered into phase II trials because of its toxicity profile. Nevertheless, JM-40 has contributed to a better understanding of possible relationships between chemical, pharmacokinetic and clinical properties of platinum compounds.

This study was supported by a grant from the Netherlands Cancer Foundation (KWF) nr. AUKC VU 83-7. F.J. Varossieau is acknowledged for technical assistance. It was performed as part of the program of the EORTC Pharmacokinetics and Metabolism Group.

### References

BENET, L.Z. & GALEAZZI, R.L. (1979). Noncompartmental determination of the steady-state volume of distribution. *J. Pharm. Sci.*, **68**, 1071.

BOVEN, E., VAN DER VIJGH, W.J.F., NAUTA, M.M., SCHLUPER, H.M.M. & PINEDO, H.M. (1985). Comparative activity and distribution studies of five platinum analogues in nude mice bearing human ovarian carcinoma xenografts. *Cancer Res.*, **45**, 86.

CLEARE, M.J., HYDES, P.C., MALERBI, B.W. & WATKINS, D.M. (1978). Anti-tumour platinum complexes: Relationships between chemical properties and activity. *Biochimie*, **60**, 835.

COLLIER, P.S. (1983). Some considerations on the estimation of steady state apparent volume of distribution and the relationships between volume terms. *J. Pharmacokin. Biopharm.*, **11**, 93.

COLLINS, J.M., ZAHARKO, D.S., DEDRICK, R.L. & CHABNER, B.A. (1986). Potential roles for preclinical pharmacology in phase I clinical trials. *Cancer Treat. Rep.*, **70**, 73.

CUTBUSH, S.D., KURODA, R. & NEIDLE, S. (1983). The antitumour complex ethylenediamine platinum(II) malonate: X-ray structure analysis, and studies of its stability in solution. *J. Inorg. Biochem.*, **18**, 213.

ELFERINK, F., VAN DER VIJGH, W.J.F., KLEIN, I., VERMORKEN, J.B., GALL, H.E. & PINEDO, H.M. (submitted). Pharmacokinetics of diammine(1,1-cyclobutanedicarboxylato)-platinum(II) (carboplatin) after intravenous administration. *Cancer Treat. Rep.*

HARLAND, S.J., NEWELL, D.R., SIDDIK, Z.H., CHADWICK, R., CALVERT, A.H. & HARRAP, K.R. (1984). Pharmacokinetics of *cis*-diammine-1,1-cyclobutane dicarboxylate platinum(II) in patients with normal and impaired renal function. *Cancer Res.*, **44**, 1693.

JOSS, R.A., KAPLAN, S., GOLDHIRSCH, A., SESSA, C., BRUNNER, K.W. & CAVALLI, F. (1984). A phase I trial of *cis*-diammine-1,1-cyclobutane dicarboxylate platinum II (carboplatin, CBDCA, JM-8) with a single dose every five week-schedule. *Invest. New Drugs*, **2**, 297.

KING, F.G., DEDRICK, R.L. & FARRIS, F.F. (1986). Physiological pharmacokinetic modeling of *cis*-dichlorodiammineplatinum(II) (DDP) in several species. *J. Pharmacokin. Biopharm.*, **14**, 131.

KOVACH, J.S. (1983). Pharmacokinetic studies of anticancer agents during phase I trials. In *Pharmacokinetics of Anticancer Agents in Humans*, Ames, M.M. *et al.* (eds) p. 433. Elsevier: Amsterdam.

LELIEVELD, P., VAN DER VIJGH, W.J.F., VELDHUIZEN, R.W. & 4 others (1984). Preclinical studies on toxicity, antitumour activity and pharmacokinetics of *cis*-platin and three recently developed derivatives. *Eur. J. Cancer Clin. Oncol.*, **20**, 1087.

LONG, D.F., PATTON, T.F. & REPTA, A.J. (1981). Platinum levels in human erythrocytes following intravenous administration of *cis*-platin: Importance of erythrocytes as a distribution site for platinum species. *Biopharm. Drug Dispos.*, **2**, 137.

METZLER, C.M., ELFRING, G.L. & MCEWAN, A.J. (1974). A package of computer programs for pharmacokinetic modeling. *Biometrics*, **30**, 562.

NEIDLE, S., ISMAIL, I.M. & SADLER, P.J. (1980). The structure of the antitumour complex *cis*(diammino)(1,1-cyclobutanedicarboxylato)Pt(II): X-ray and nmr studies. *J. Inorg. Biochem.*, **13**, 205.

REECE, P.A., STAFFORD, I., RUSSEL, J. & GILL, P.G. (1986). Reduced ability to clear ultrafilterable platinum with repeated courses of *cis*-platin. *J. Clin. Oncol.*, **4**, 1392.

REPTA, A.J. & LONG, D.F. (1980). Reactions of *cis*-platin with human plasma and plasma fractions. In *Cisplatin, Current Status and New Developments,* Prestayko, A.W. *et al.* (eds) p. 285. Academic Press: New York.

ROSE, W.C. & BRADNER, W.T. (1984). Experimental antitumor activity of platinum coordination complexes. In *Platinum Coordination Complexes in Cancer Chemotherapy*, Hacker, M.P. *et al.* (eds) p. 228. Martinus Nijhoff: Boston.

SCHURIG, J.E., FLORCZYK, A.P. & BRADNER, W.T. (1984). Evaluation of platinum complexes for emetic potential. In *Platinum Coordination Complexes in Cancer Chemotherapy*, Hacker, M.P. *et al.* (eds) p. 187. Martinus Nijhoff: Boston.

VAN DER VIJGH, W.J.F., LELIEVELD, P., KLEIN, I., VAN PUTTEN, L.M. & PINEDO, H.M. (1983). Pharmacokinetics of five platinum compounds in dogs. In *Proc. 13th Int. Congress of Chemotherapy, New Drugs in Cancer Chemotherapy*, Spitzy, K.H. & Karrer, K. (eds) p. 286/57. Vienna.

VAN DER VIJGH, W.J.F., ELFERINK, F., POSTMA, G.J., VERMORKEN, J.B. & PINEDO, H.M. (1984). Determination of ethylenediamine-platinum(II) malonate in infusion fluids, human plasma and urine by high performance liquid chromatography. *J. Chromatogr.*, **310,** 335.

VAN DER VIJGH, W.J.F. & KLEIN, I. (1986). Protein binding of five platinum compounds. Comparison of two ultrafiltration systems. *Cancer Chemother. Pharmacol.*, **18,** 129.

VAN HENNIK, M.B., VAN DER VIJGH, W.J.F., KLEIN, I. & 4 others (1987). Comparative pharmacokinetics of *cis*-platin and three analogs in mice and man. *Cancer Res.* (in press).

VERMORKEN, J.B., TEN BOKKEL HUININK, W.W., McVIE, J.G., VAN DER VIJGH, W.J.F. & PINEDO, H.M. (1984*a*). Clinical pharmacology of *cis*-platin and some new platinum analogs. In *Proc. 2nd World Conference on Clinical Pharmacology and Therapeutics*, Lemberger, L. & Reidenberg, M.M. (eds) p. 967. *Am. Soc. Pharmacol. Ther.*

VERMORKEN, J.B., VAN DER VIJGH, W.J.F., KLEIN, I., HART, A.A.M., GALL, H.E. & PINEDO, H.M. (1984*b*). Pharmacokinetics of free and total platinum species after short-term infusion of *cis*-platinum. *Cancer Treat. Rep.*, **68,** 505.

VERMORKEN, J.B., WINOGRAD, B. & VAN DER VIJGH, W.J.F. (1985). Clinical Pharmacology of *cis*-platin and some new platinum analogs. In *Proc. 14th Congress of Chemotherapy. Recent advantages in chemotherapy; anticancer section,* Ishigami, J. (ed) p. 96. University of Tokyo Press: Tokyo.

VERMORKEN, J.B., VAN DER VIJGH, W.J.F., KLEIN, I. & 4 others (1986). Pharmacokinetics of free and total platinum species after rapid and prolonged infusions of *cis*-platin. *Clin. Pharmacol. Ther.*, **39,** 136.

WAGNER, J.G. (1976). Linear pharmacokinetic equations allowing direct calculation of many needed pharmacokinetic parameters from the coefficients and exponents of polyexponential equations which have been fitted to the data. *J. Pharmacokin. Biopharm.*, **4,** 443.

WAGNER, J.G. & coworkers (1977). Pharmacokinetic parameters estimated from intravenous data by uniform methods and some of their uses. *J. Pharmacokin. Biopharm.*, **5,** 161.

WINOGRAD, B., VERMORKEN, J.B., TEN BOKKEL HUININK, W.W. & 6 others (1986). Phase I study of ethylenediamine platinum(II) malonate (NSC 146068), a second generation platinum analogue. *Cancer Res.*, **46,** 2148.